# Ultrasound and Microbubbles for the Treatment of Ocular Diseases: From Preclinical Research towards Clinical Application

**DOI:** 10.3390/pharmaceutics13111782

**Published:** 2021-10-25

**Authors:** Charis Rousou, Carl C. L. Schuurmans, Arto Urtti, Enrico Mastrobattista, Gert Storm, Chrit Moonen, Kai Kaarniranta, Roel Deckers

**Affiliations:** 1 Departments of Pharmaceutics, Utrecht Institute for Pharmaceutical Sciences, Utrecht University, Heidelberglaan 8, 3584 CS Utrecht, The Netherlands; c.c.l.schuurmans@uu.nl (C.C.L.S.); e.mastrobattista@uu.nl (E.M.); g.storm@uu.nl (G.S.); 2 Division of Imaging and Oncology, University Medical Center Utrecht, Heidelberglaan 100, 3584 CX Utrecht, The Netherlands; c.moonen@umcutrecht.nl (C.M.); r.deckers-2@umcutrecht.nl (R.D.); 3 Department of Pharmacology, Utrecht Institute for Pharmaceutical Sciences, Utrecht University, Heidelberglaan 8, 3584 CS Utrecht, The Netherlands; 4 School of Pharmacy, Faculty of Health Sciences, University of Eastern Finland, 70210 Kuopio, Finland; arto.urtti@uef.fi; 5 Division of Pharmaceutical Biosciences, Faculty of Pharmacy, University of Helsinki, P.O. Box 56, 00014 Helsinki, Finland; 6 Institute of Chemistry, St. Petersburg State University, Universitetskii Pr. 26, Petrodvorets, 198504 St. Petersburg, Russia; 7 Department of Biomaterials Science and Technology, University of Twente, 7500 AE Enschede, The Netherlands; 8 Department of Surgery, Yong Loo Lin School of Medicine, National University of Singapore, Singapore 119228, Singapore; 9 Department of Ophthalmology, Kuopio University Hospital, P.O. Box 100, 70029 Kuopio, Finland; kai.kaarniranta@uef.fi; 10 Department of Ophthalmology, Institute of Clinical Medicine, University of Eastern Finland, P.O. Box 1627, 70211 Kuopio, Finland

**Keywords:** ultrasound, microbubble, drug delivery, blood–retina barrier, ocular drug delivery, USMB, cellular drug delivery

## Abstract

The unique anatomy of the eye and the presence of various biological barriers make efficacious ocular drug delivery challenging, particularly in the treatment of posterior eye diseases. This review focuses on the combination of ultrasound and microbubbles (USMB) as a minimally invasive method to improve the efficacy and targeting of ocular drug delivery. An extensive overview is given of the in vitro and in vivo studies investigating the mechanical effects of ultrasound-driven microbubbles aiming to: (i) temporarily disrupt the blood–retina barrier in order to enhance the delivery of systemically administered drugs into the eye, (ii) induce intracellular uptake of anticancer drugs and macromolecules and (iii) achieve targeted delivery of genes, for the treatment of ocular malignancies and degenerative diseases. Finally, the safety and tolerability aspects of USMB, essential for the translation of USMB to the clinic, are discussed.

## 1. Introduction

Ophthalmic ultrasound as a research field started in 1956, when researchers Mundt and Hughes, using an ultrasonic reflectoscope, found that they could calculate the horizontal distance between ocular tissues based on sent ultrasound waves and received echoes in a single axis (a technique known as A-mode ultrasonography) [1]. Since then, ocular ultrasound has flourished, and became one of the standard imaging techniques clinically applied in ophthalmology. Some examples of modern ophthalmic ultrasound applications in the clinic include the diagnosis of intraocular tumors, the detection of structural changes in glaucoma and retinal detachment and the use of high frequency ultrasound (35–70 MHz) to construct high resolution images of the anterior eye (a method known as ultrasound biomicroscopy, UBM) [2,3,4].

Around the same time (mid-1960s), microbubbles made their entrance into the ultrasound field [5]. Microbubbles are gas-filled spheres with a diameter typically between 0.5 and 10 μm [6]. They are intravenously administered and used as ultrasound contrast agents as they improve blood-to-tissue contrast during ultrasound imaging. Microbubbles dissolve in blood after injection and, if the vasculature is intact, they circulate in the bloodstream until they are eliminated by exhalation or phagocytosis [7,8]. Microbubble-specific imaging methods, so-called contrast enhanced ultrasound (CEUS), have been developed and are currently used as a diagnostic tool in the clinic. An in-depth explanation of the diagnostic applications of CEUS can be found elsewhere [7,9]. Microbubbles first entered the field of ophthalmology in 1994, to enhance the blood flow signal in ocular and orbital malignancies [10,11]. Other clinical examples of CEUS in ophthalmology include imaging of the microcirculation in benign ocular lesions, differentiation between subretinal hemorrhage and hypovascular tumors, and quantification of perfusion-specific parameters [12,13,14,15]. Extensive reviews on the different microbubble imaging methods used in ophthalmology can be found elsewhere [4,16,17,18].

**Figure 1:**
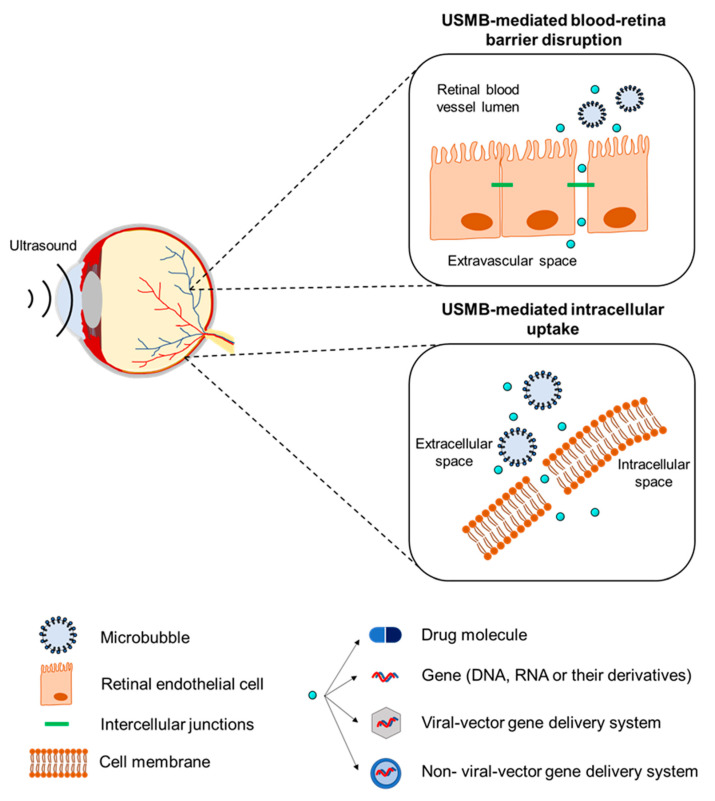
Schematic illustration of the therapeutic applications of USMB.

Microbubbles can also be used in therapeutic ultrasound applications. Microbubbles exposed to ultrasound waves undergo oscillations that induce various bioeffects on the surrounding structures. These bioeffects can be exploited to improve the local administration of drugs and genes. Recently, preclinical investigations of the therapeutic use of ultrasound and microbubbles (USMB) have been conducted in the field of ophthalmology, where there is a clear need for improved new delivery methods. The aim of this review is to provide an overview of the different therapeutic applications of USMB in ophthalmology (Figure 1). These can be divided into two distinct applications: (i) disruption of the blood–retina barrier and extravasation of drugs/genes that circulate in the bloodstream, and (ii) intracellular uptake of drugs/genes in various ocular cells.

The structure of this review is as follows: first, a brief introduction on the mechanisms that underlie USMB-mediated therapy is given (Section 2), followed by an overview of the effect of biological barriers on the pharmacokinetics of ocular drug delivery (Section 3). Various studies that investigated the application of USMB in the treatment of different ocular diseases are presented in Section 4. Finally, safety and tolerability aspects important in the clinical translation of USMB (Section 5) and future directions (Section 6) are discussed.

## 2. Mechanisms Underlying the Therapeutic Use of Ultrasound and Microbubbles

Microbubbles exposed to ultrasound waves will alternate between contraction and expansion due to their compressible nature. The amplitude of microbubble oscillation depends on the amplitude of the ultrasound pressure wave. Ultrasound amplitude may be expressed as acoustic pressure (measured in MPa), intensity (measured in W/cm^2^), or mechanical index (MI). The latter is a unitless parameter defined as the ratio of the peak negative pressure (PNP, in MPa) over the square root of the transmitted ultrasound frequency (in MHz). A microbubble responds with linear oscillations at low ultrasound pressures. The microbubble starts oscillating asymmetrically with increased pressure, as the absolute microbubble radius change during expansion is larger than during contraction. This phenomenon is known as stable or non-inertial microbubble cavitation. Further increases in ultrasound pressure cause the microbubble to oscillate more violently, until it becomes unstable and collapses (also known as inertial microbubble cavitation).

Different biophysical events can occur depending on the oscillation regime of the microbubbles, which will have various effects on cells in close proximity. During stable cavitation, a microbubble pushes and pulls the membrane of the adjacent cell as a result of microbubble expansion and contraction, respectively. Furthermore, mechanical forces are developed on the cell membrane induced by microstreaming formation around the oscillating microbubble. Finally, inertial cavitation is associated with the formation of shock waves and liquid micro-jets that act as micro-syringes on the cell membrane during microbubble collapse [19,20]. These biophysical events can, in turn, lead to various bioeffects on the cells. Particularly interesting are the bioeffects induced by stable and inertial cavitation, as they are potent in (i) enhancing the membrane permeability of cells in close vicinity and improving the intracellular uptake of drugs, (ii) increasing the permeability of blood vessel linings and allowing for extravasation of drugs, and (iii) altering the flow of blood inside blood vessels.

**Figure 2:**
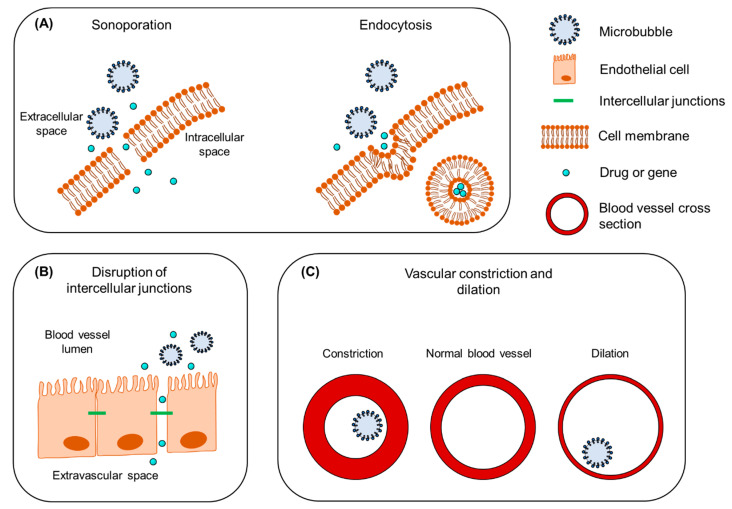
The mechanisms underlying the therapeutic use of USMB. (**A**) Intracellular uptake of drug/gene induced by sonoporation (**left**) and endocytosis (**right**). (**B**) Disruption of intercellular junctions and extravasation of drug/gene from blood vessel to the extracellular space. (**C**) Vascular constriction and dilation. Figure not to scale.

The mechanical stresses caused by an oscillating or collapsing microbubble lead to the formation of pores in the cell membrane (Figure 2A), so-called sonoporation (or sonopermeation). It has been shown that pore formation and the resulting intracellular uptake of molecules occur immediately or within a few minutes after USMB treatment [21,22,23]. USMB may also stimulate endocytosis (Figure 2A), though the mechanisms underlying this bioeffect are not yet fully understood [21]. Sonoporation and endocytosis can induce the uptake of molecules (fluorescent dextrans, calcein, propidium iodide, liposomes, plasmid DNA) that are otherwise not able to permeate intact cell membranes, because of their large size and/or low lipophilicity [24,25,26,27,28]. Similar to stable cavitation, sonoporation induced by inertial cavitation has been exploited to enhance the uptake of drugs and genes by cells [29,30,31,32]. Whether inertial cavitation can safely induce endocytosis still remains to be investigated. The pore size as a result of sonoporation highly depends on the microbubble cavitation regime (stale or inertial): low PNP induces pores with sizes from several tens to a few hundred nanometers, while high PNP results in pores with sizes of a few micrometers [21,33]. Similar to the size of pores, the kinetics of membrane resealing are defined by the acoustic pressure used and can vary between milliseconds and minutes [21]. Notably, the bioeffects caused by stable cavitation do not negatively affect cell viability, while the formation of larger pores correlates with reduced cell viability as a result of apoptosis [33,34].

The endothelium in blood capillaries, with a diameter in the range of the size of microbubbles (~5 μm), experiences mechanical forces exerted by the cavitating microbubbles. This mechanical activity increases the permeability of the endothelium by influencing the cytoskeleton arrangement in the endothelial cells and altering the expression of intercellular junction proteins (Figure 2B) [21,35]. A widely studied application of this USMB-induced bioeffect is the temporal disruption of the blood–brain barrier (BBB) and deposition/extravasation of drug molecules in the brain parenchyma [36,37]. In contrast to sonoporation-induced intracellular uptake, increases in the paracellular diffusion of compounds via intercellular gaps were found to be prolonged, on the time scale of several hours [35,38,39]. Preclinical studies have shown that the extent of BBB disruption is highly dependent on the frequency of the transmitted ultrasound waves, PNP, and exposure time [40,41]. An in vivo animal study demonstrated that, for various PNPs, the opening size of tight junctions in the mouse brain endothelium ranged between 2 nm and approximately 50 nm [42]. It was previously observed that stable cavitation can induce BBB permeability without inducing side effects, while inertial cavitation induces both barrier disruption and extravasation of erythrocytes as a result of the more violent bioeffects and larger opening size [43]. Compared with sonoporation, less information is available on the kinetics of tight junction opening and resealing, due to the lack of adequate technology for microscopic imaging of the interaction between microbubbles and vascular endothelium [21].

A third mechanism induced by USMB is the alteration of blood flow inside the blood vessels. As a consequence of cavitating microbubbles activity in the vasculature, two contradictory phenomena have been reported: vascular invagination or shutdown and vascular dilation (Figure 2C). Chen et al. studied the behavior of cavitating microbubbles (11 MPa PNP) in ex vivo microvessels using high-speed microscopy [44]. In a microvessel where microbubbles were in contact with the endothelium, microbubbles initially expanded, causing microvessel dilation, followed by immediate microbubble collapse and microvessel invagination (constriction). Immediately afterwards, microbubble remnants were observed outside the microvessel walls, indicating blood vessel rupture. In line with this study, Hwang et al. observed a positive correlation between inertial cavitation (1–9 MPa PNP) and blood vascular damage in vivo [45]. This microbubble-driven blood vessel narrowing might explain the significant decrease in blood perfusion previously observed in numerous preclinical studies [46,47,48,49,50,51]. On the other hand, some studies have shown that USMB can induce blood vessel dilation and local increase in blood perfusion [50,52,53]. This phenomenon is known as vasodilation and could be of great importance in the treatment of postischemic cardiac damage [52]. In their study, D’Souza et al. treated hepatocellular carcinoma-bearing rats with two different USMB doses [50]. The high USMB dose was at an intensity of 2.0 W/cm^2^, with 6 min exposure time. In the low USMB dose group, ultrasound intensity and exposure time were reduced by half. Quantification of perfusion indicated a decrease in blood flow as a result of vascular disruption in the group that received the high-dose therapy. In contrast, the low USMB dose group showed an enhancement in tumor blood perfusion, but this result was not consistent. This study demonstrated that ultrasound settings and microbubble dose might be critical factors in balancing between USMB-induced vascular dilation and constriction. More extensive information on the mechanisms that underlie USMB-mediated drug delivery and the induced bioeffects can be found elsewhere [21,31,33].

## 3. The Effect of Biological Barriers on the Pharmacokinetics of Ocular Drug Delivery

**Figure 3:**
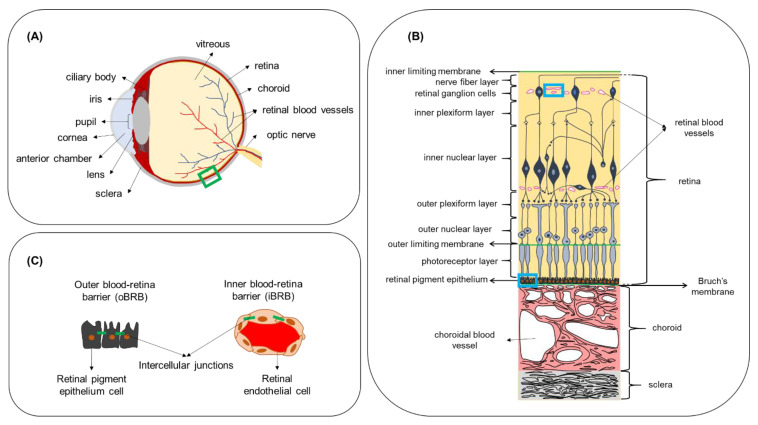
(**A**) Anatomy of the eye. (**B**) Boxed region (green line) in (**A**) showing a cross-section of the posterior eye with the different cell layers of the retina, Bruch’s membrane, choroid and sclera. (**C**) Boxed regions in (**B**) (blue line) showing the locations where the outer and inner blood–retina barriers are formed, due to intercellular junctions between adjacent cells. Figure not to scale.

The eye is a unique sensory organ in terms of anatomy, physiology, and function. Anatomically, it can be divided into anterior and posterior segments (Figure 3A). The anterior eye consists of the tissues in the front third of the eyeball, which include the cornea, conjunctiva, anterior sclera, iris, ciliary body, lens and the anterior chamber. The posterior eye consists of the vitreous humor, neural retina, Bruch’s membrane, choroid and posterior sclera. The retina can be subdivided into the neural retina and the retinal pigment epithelium (RPE) (Figure 3B). The retina is a semi-transparent tissue constructed by multiple neural cells in laminar arrangement. The RPE is an epithelial cell monolayer that is pigmented due to the presence of melanin. The main role of RPE is to phagocytose debris and waste products from photoreceptors and, as discussed below, to form the outer blood–retina barrier. Bruch’s membrane, an extracellular matrix rich in collagen and elastin, is located between the RPE and choroidal capillaries and the choroid (a network of blood vessels). More detailed information about ocular physiology and the function of the different neural retinal cell types can be found elsewhere [54,55,56].

Due to the unique anatomy of the eye, i.e., the presence of the ocular barriers, the development of efficacious ocular therapeutics poses a major challenge in ophthalmology, especially in the treatment of retinal diseases. Here, we briefly discuss some of the main routes of drug administration in the eye and the main drawbacks of current treatments. Topical administration of eye drops is a traditional therapeutic modality for the treatment of anterior eye diseases such as inflammations and increased intraocular pressure (IOP), such as in the treatment of glaucoma. It is characterized by high patient compliance, as eye drops can easily be self-administered daily. However, the bioavailability of topically administered drugs is between 1 and 4% due to the rapid drainage from the surface of the eye and systemic absorption of fluids by the conjunctiva, making this delivery method unsuitable for the treatment of posterior eye diseases [57]. After intravitreal administration, drugs are eliminated by the anterior or posterior route [58]. Drug molecules first diffuse to the posterior vitreous chamber, then via outflow channels in the trabecular meshwork, and they are eliminated by the aqueous humor (anterior route). If a drug is capable of crossing the endothelia of the BRB and blood–aqueous barrier (BAB), i.e., it is smaller than 2 nm in size, it is eliminated via the posterior route. Generally, the half-life of intravitreally administered small molecules is between 1 to 10 h, and several days for larger molecules such as proteins [59,60], creating a need for repeated injections. Periocular administration includes the injection of drug molecules in locations around the eyeball, and it results in improved bioavailability of drugs in the anterior tissues as compared to topical administration [58]. However, the retinal bioavailability of a small molecule is limited to only 0.1%, underscoring the challenge associated with replacing intravitreal with subconjunctival injections, despite the fact that the subconjunctival route is less invasive and less associated with adverse effects such as retinal detachment and increase in IOP [58,61].

In the human eye, the exchange of nutrients for normal function of the neural cell layers in the retina is maintained by two independent vascular networks: the retinal vasculature and the choroid. To protect the retina from harmful substances present in the blood circulation and to regulate the exchange of molecules with the bloodstream in a controlled manner, a specialized barrier function is situated between the vascular endothelium and epithelium of the retina. This retinal barrier resembles the BBB in terms of function and ultrastructure. Junctional complexes (tight and adherens junctions) are present at the level of the RPE and retinal endothelium and form the outer blood–retina barrier (oBRB) and inner blood–retina barrier (iBRB), respectively (Figure 3C). The iBRB allows only molecules that are smaller than 2 nm to freely permeate, such as mannitol (molecular radius—0.4 nm [62]) and carboxyfluorescein (molecular radius—0.5 nm [63]) [58]. The permeability of molecules across the oBRB is highly dependent on their hydrophilicity. Indicatively, the apparent permeability coefficient for betaxolol (LogD 1.59; M_W_ 307 Da) was calculated in ex vivo bovine RPE-choroid to be 16.7 × 10^−6^ cm/s, while the corresponding value for carboxyfluorescein (LogD -3.15; M_W_ 376 Da) was 0.96 × 10^−6^ cm/s, showing that the higher the hydrophilicity, the less the transport across the oBRB [64]. While the presence of these natural barriers protects the eye from the invasion of foreign substances and regulates the environment of ocular tissues, it also hinders the delivery of therapeutics in the case of ocular disease. Intravenous administration for ocular drug targeting is generally characterized by higher patient compliance compared to intraocular injections, as it is a less invasive method. Additionally, it is not associated with increased IOP and intraocular hemorrhage, retinal detachment or vitreous hemorrhage or other side effects commonly associated with intravitreal injections [65,66,67]. In addition to the iBRB and oBRB, another biological factor that regulates drug transfer from the bloodstream to the retina is the high blood flow in the choroid relative to the tissue (peak systolic velocity of about 10 mm/s measured in healthy human eyes [68]). However, quantitative information about retinal bioavailability after intravenous administration is not available. Some physiological characteristics, such as fenestrations in choriocapillaries with size 70–80 nm [58], and the active RPE internalization of compounds such as low-density lipoprotein [69], make targeted retinal drug delivery from the bloodstream an interesting drug delivery approach.

## 4. Ocular Pathologies That Could Benefit from Therapeutic Ultrasound and Microbubbles

In this section, we summarize the studies that investigated the use of USMB to overcome the biological barriers discussed in the previous section and enhance, thereby, the ocular delivery of drugs and genes for the treatment of various ocular diseases (Table 1). In each subsection, we provide information about the pathologic characteristics of each disease, along with the most frequently used drug treatment methods and the associated challenges. Finally, we discuss the main findings from in vitro and in vivo animal studies reported in the literature and how these can contribute to improve ocular drug delivery.

### 4.1. Wet Age-Related Macular Degeneration

**Figure 4:**
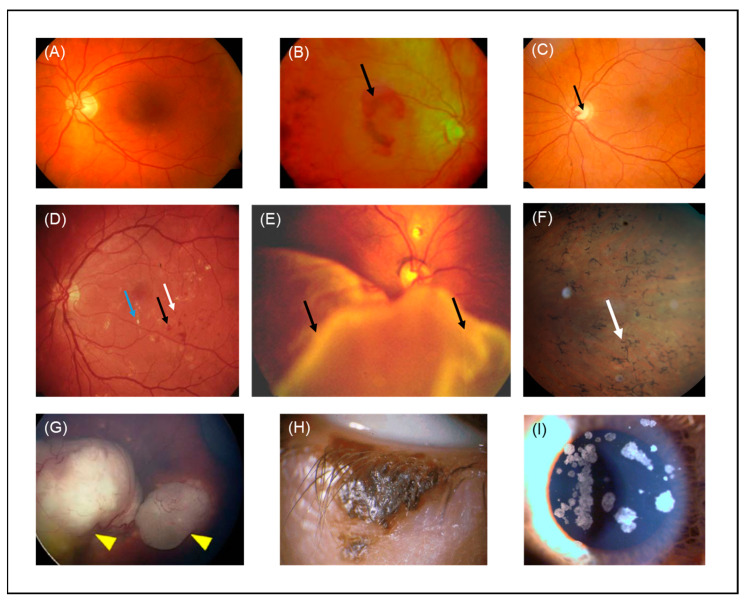
Color fundus photographs of (**A**) a healthy individual, (**B**) a patient with wet AMD (black arrow indicates hemorrhage, (**C**) a patient with glaucoma (black arrow indicates optic disc excavation), (**D**) a patient with DR (blue, black and white arrows indicate exudation, hemorrhage and microaneurysm, respectively), (**E**) a patient with retinal detachment (black arrows indicate PVR), (**F**) a patient with RP (white arrow indicates bone-spicule deposits), (**G**) a patient with RB (yellow arrowheads indicate tumors) adapted with permission from [148], John Wiley and Sons, 2018, (**H**) a patient with eyelid malignant melanoma. (**I**) Slit lamp image of a granular cornea dystrophy from a patient with corneal opacities. AMD: age-related macular degeneration, DR: diabetic retinopathy, PVR: proliferative vitreoretinopathy, RP: retinitis pigmentosa, RB: retinoblastoma.

Age-related macular degeneration (AMD) is a progressive chronic disease of the neural retina and choriocapillaris caused by the deposition of acellular polymorphous debris (drusen) between the RPE and Bruch’s membrane. Histochemical characterization of drusen revealed that they are composed of a mucopolysaccharide (sialomucin) and a cerebroside lipid [70]. Tissue metalloproteinase inhibitor 3, clusterin, vitronectin, serum albumin, crystallin and complement proteins are rich in AMD drusen [71]. Drusen develop with age as a result of RPE degeneration and their size is used to determine the grade of AMD [72]. Advanced AMD is classified into (i) non-neovascular/dry/atrophic, which is characterized by geographic atrophy and drusen formation that extend to the center of the macula, and (ii) neovascular/wet/exudative, where choroidal neovascularization is additionally present. Another clinical hallmark of wet AMD is retinal hemorrhage (Figure 4B).

Currently, the most common treatment method for wet AMD is intravitreal injection of anti-vascular endothelial growth factor (anti-VEGF) drugs. The first Food and Drug Administration (FDA)-approved anti-VEGF was Pegaptanib (Macugen, Pfizer), an oligonucleotide drug that binds the VEGF-163 isoform, followed by ranibizumab (Lucentis, Genentech/Novartis), an antibody fragment that binds all VEGF isoforms. Bevacizumab (Avastin, Genentech) is a full-length antibody that binds all VEGF isoforms that are currently approved for systemic malignancies; however, it is also used off-label for the treatment of wet AMD. Aflibercept (VEGF Trap-Eye, Regeneron/Bayer) is a recently FDA-approved engineered protein that binds VEGF [73]. The last addition to anti-VEGF treatments for wet AMD is brolucizumab [74]. The limited half-lives of anti-VEGF drugs after intravitreal administration (9 days for ranibizumab and about 5 days for bevacizumab, estimated 7 days for aflibercept [75,76,77], and about 2.5 days for brolucizumab [78]) create a need for repeated injections (monthly or bi-monthly), which are frequently associated with patient discomfort due to injection-related pain and adverse effects (e.g., hemorrhages, retinal detachment, endophthalmitis) [58,76]. Intravenous administration, on the other hand, is a less invasive and more patient-compliant delivery method; however, this approach is currently limited by the low permeability of the BRB for many drugs. Considering the functional and structural similarities between the BBB and BRB, USMB has the potential to enhance the efficacy of intravenously administrated drugs against AMD. In addition, other ocular disorders with the pathology site in close proximity with the BRB, such as diabetic retinopathy or retinoblastomas, may also benefit from USMB-induced BRB disruption.

**Figure 5:**
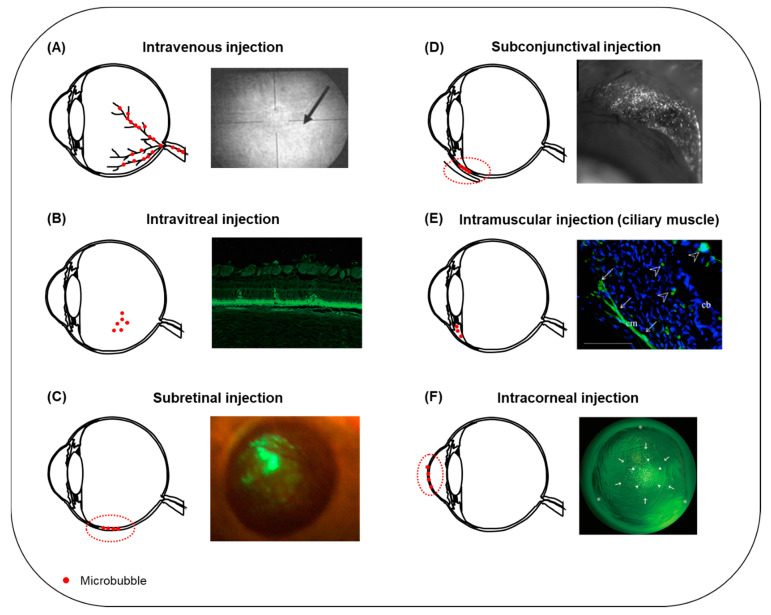
Summary of microbubble injection sites reported in the literature for USMB-mediated ocular drug and gene delivery. (**A**) Following intravenous administration, microbubbles circulate in the retinal blood vessels (**left**). Microbubble oscillations can induce disruption of the BRB and extravasation of fluorescein from blood retinal vessels (**right**, arrow). Adapted with permission from [79], John Wiley and Sons, 2007. (**B**) The most frequently encountered administration site for microbubbles in ocular drug delivery is intravitreal injection (**left**). Fluorescence microscopy image of a rabbit retinal section after treatment with bubble liposomes in combination with plasmid GFP and intravitreal ultrasound (**right**). GFP-positive cells were present in the ONL. Adapted from [85] Hindawi, 2012. (**C**) Schematic illustration of microbubbles injected into the subretinal space (**left**, dashed line). Fluorescent fundus stereoscopic image of rat retina treated with rAAV2-EGFP and USMB (**right**). GFP signal covered a large retinal surface area and was visible for 120 days post-treatment. Adapted from [87], Sciendo, 2009. (**D**) Schematic illustration of subconjunctival injection of microbubbles (**left**, dashed line). Expression of GFP on the conjunctiva of a rat 48 h after treatment with plasmid-GFP combined with bubble liposomes and ultrasound (**right**). Adapted with permission from [103], Elsevier, 2007. (**E**) Schematic illustration of microbubbles injected into the ciliary muscle (**left**). Fluorescence microscopy image of a rat eye after combined treatment with pEGFP-C1 gene, intramuscular microbubbles and ultrasound (**right**). GFP-positive cells were localized in the ciliary muscle (arrows) and the ciliary body (arrowhead). Adapted with permission from [112], Elsevier, 2011. (**F**) USMB-mediated drug or gene delivery in the cornea can be facilitated upon intracorneal injection of microbubbles (**left**, dashed line). Fluorescence stereomicroscopic image of a rabbit cornea, 7 days post-treatment with plasmid GFP and USMB (**right**). A mixture of microbubbles and plasmid was injected into the center of cornea (arrows), and ultrasound treatment was performed with the ultrasound probe positioned on the corneal surface (arrowheads). GFP-positive cells were colocalized with the area where the ultrasound probe was positioned. Adapted with permission from [147], ARVO, 2006. Figure not to scale.

The first study to determine the feasibility of BRB opening using USMB was performed by Hirokawa et al. in 2007 [79]. Rabbit eyes were treated with USMB at a frequency of 2 MHz, at two different MIs (0.2 and 1.7). Comparison between fluorescein fundus angiography and fundus photography images acquired pre- and post-treatment revealed alteration in the diameter of the uveal blood vessels in 20% of the eyes treated at low MI. In contrast, 80% of the eyes treated at high MI had altered uveal vessels. In addition, the average retinal vein (but not the artery) diameter was significantly reduced in the high MI-treated eyes after treatment, suggesting the occurrence of vasoconstriction. At high MI, leakage of fluorescein (M_W_ 332 Da) was observed in the fundus of one eye, suggesting increased permeability of blood vessels (Figure 5A). No bleeding was observed, though microscopic hemorrhage could not be excluded. Similarly, Park et al. investigated the effect of USMB on BRB permeability in rat eyes by investigating the extravasation of gadolinium (Gd, M_W_ 938 Da) [80]. USMB treatment was performed at 0.69 MHz frequency and three different pressures (0.81, 0.88 and 1.10 MPa, corresponding to MI 0.98, 1.06 and 1.32, respectively). Quantification of Gd-enhanced magnetic resonance images revealed that signal intensity increased immediately after USMB treatment for all three MIs and was the highest for 1.1 MPa, indicating disruption of the BRB. A second Gd injection 3.5 h after treatment did not result in signal enhancement, showing for the first time that this BRB disruption was reversible at 0.81 and 0.88 MPa, but not at 1.10 MPa. Retinal microscopic examination revealed the presence of extravasated erythrocytes in the nuclear retinal layer 24 h after treatment. Most retinal damage was observed at the highest pressure. Touahri et al. investigated the effect of USMB on the extravasation of macromolecules with different sizes: Evans blue (M_W_ 66 kDa after binding to serum albumin), immunoglobulin G (IgG) monomer (M_W_ 150 kDa) and immunoglobulin M (IgM) pentamer (M_W_ 970 kDa) [81]. BRB disruption was investigated in rats at MIs ranging between 0.3 and 0.8, and frequency 1.1 MHz. In five out of six rats, all three macromolecules were localized in the deep and superficial vascular plexi (INL and RGC layers) as a result of iBRB permeabilization. In contrast, no evidence indicating extravasation from the choroidal plexus and penetration of molecules to the neural retina through the oBRB was found. Evidence for neuroinflammation and presence of erythroid cells was found in 33% of the eyes, 30 min after treatment with USMB, and co-localization with Evans blue was observed. Hematoxylin and eosin (H&E) staining of retinal sections revealed no morphological alterations in 83% of the animals.

In addition to increasing the permeability of the BRB, USMB can also increase the permeability of plasma membranes and increase the intracellular concentration of drug molecules. Numerous research groups have investigated the effect of USMB in the intracellular uptake of macromolecules [82], genes [83,84,85,86,87,88,89] and nanoparticles [90,91] by the neural retina and RPE. Although these studies are discussed here as drug delivery applications against AMD, the same principles could be applied for the treatment of other retinal diseases discussed later in this section, such as retinitis pigmentosa.

Thakur et al. studied the intracellular delivery of a model IgG in three retinal cell lines (human RPE cells and human-derived Müller glia and mouse-derived photoreceptors) [82]. These in vitro experiments were conducted using custom-made nanobubbles (NBs) activated by ultrasound at a frequency of 1 MHz. They found that ultrasound in combination with nanobubbles (USNB) significantly increased the uptake of the macromolecule in retinal cells compared with ultrasound only or USMB (using conventional microbubbles). However, the increase in the intracellular uptake was significant only for the two human-derived cell lines, showing that induced effects are cell line-dependent. Furthermore, shorter exposure time in combination with higher ultrasound intensity (20 s at 1.0 W/cm^2^ vs. 30 s at 0.5 W/cm^2^) had a more significant effect on intracellular uptake, showing that USNB efficacy is highly dependent on these two ultrasound parameters.

The effect of ultrasound parameters in the intracellular uptake of macromolecules/genes in retinal cells was also investigated by Wan et al. [83]. Specifically, they determined how ultrasound intensity, exposure time and the ratio of microbubbles to cells affected cell viability and intracellular uptake of a positively charged complex (PEI/pEGFP). They observed that increased ultrasound intensity resulted in higher amounts of EGFP-positive cells but also more extensive cell damage. The exposure time was inversely proportional to cell viability. These in vitro results were confirmed in vivo by injecting PEI/pEGFP alone or combined with USMB (frequency 1 MHz, intensity 2 W/cm^2^, exposure time 5 min) subretinally in rat eyes. Examination of frozen tissues revealed that, in the group treated with USMB, 7 out of 12 retinas appeared with diffused EGFP-positive cells, mainly distributed in the neural retina. Using the same ultrasound parameters as Wan et al., Li et al. examined the differences in EGFP expression after subretinal injection of microbubbles and ultrasound treatment [84]. A significantly greater number of GFP-positive cells was found in eyes treated with plasmid + USMB compared with plasmid alone, plasmid + microbubbles and plasmid + ultrasound. GFP-signal was distributed in the neural retina and RPE. Differences in fluorescent signal between PEI/pDNA and PEI/pDNA + USMB were significant during the first 5 days post-treatment, showing that USMB enhanced and accelerated the expression of EGFP by allowing PEI/pDNA to enter the cytoplasm earlier via sonoporation.

In contrast to the studies discussed above where ultrasound was externally applied, Sonoda et al. developed a miniature ultrasound probe to be intraocularly positioned after vitrectomy in rabbit eyes [85]. Bubble liposomes (BLs) were injected intravitreally in combination with a GFP-plasmid. Fluorescence microscopy imaging revealed that the eyes treated with plasmid + USBL (frequency 3 MHz, intensity 0.15 W/cm^2^, exposure time 60 s) had 8 times greater numbers of GFP-positive cells per visual field (32.0 cells) compared with plasmid + BL (0 cells) or plasmid + ultrasound (4.2 cells). GFP-positive cells were co-localized with the sonicated area and mainly appeared in the outer nuclear area (Figure 5B). Eye physiology was examined 1 and 3 days post-treatment, with no obvious tissue damage, which was confirmed by histology. Applying ultrasound using an intraocular probe can help to reduce acoustic energy attenuation by the anterior tissues and provide more precise control of the area exposed to ultrasound compared with externally applied probes. However, it remains open for investigation whether these arguments are enough to compensate for inducing the need for vitrectomy, that, unlike externally applied ultrasound using an ocular probe, is an invasive procedure that needs to be performed by a retinal surgeon. Moreover, precise localization of the ultrasound probe is needed so that retinal injury is avoided.

The transduction efficiency of recombinant adeno-associated virus (rAAV) vectors expressing EGFP in combination with USMB was investigated in RPE cells by Li et al. and Zheng et al. [86,87]. In vitro experiments were conducted using human-derived (Li et al.) and rat-derived (Zheng et al.) RPE cells. Li et al. reported that exposure to ultrasound only was not enough to increase the number of GFP-positive cells. When USMB was applied, an increase of about 10% was observed in the RPE cells compared with ultrasound alone. On the other hand, Zheng et al. observed an increase in number of GFP-positive cells in rAAV + ultrasound (approximately 22% of control cells), but not in rAAV + USMB treatment (about 9% of control cells). This might be due to differences in the sensitivity between the two cell-lines in microbubble-induced bioeffects. Despite this difference in the in vitro studies, USMB-combined treatment with rAAV resulted in the highest expression of EGFP following subretinal administration of microbubbles in rat eyes. In both studies, the GFP signal in the rAAV + USMB group was significantly higher than in all other groups in the first 35 days after treatment. In the study of Zheng et al., GFP signal could be detected 120 days post-treatment (Figure 5C). No alterations were observed in retinal morphology in either study.

An in vivo study on the delivery of fluorescently labelled lipofectamine-formulated-siRNA (L/siRNA) in rat retinas was performed by Zheng et al. [88]. Microscopic examination of retinas performed 12 h after treatment revealed that the highest ratio of transfected cells for the group that received L/siRNA + USMB was 57.8%, compared with the group that received L/siRNA + ultrasound (19.7%), L/siRNA (12.6%) and naked siRNA + USMB (4.3%). No signs of tissue damage, inflammation or photoreceptor loss were seen twelve hours post-treatment.

Using RPE cells in vitro, Li et al. compared the improvement in efficiency of USMB-mediated transfection/transduction between different genes (pDNA, siRNA) and different delivery vectors (chemical: PEI/pDNA, L/siRNA and biological: rAAV) [89]. Cells were exposed to different USMB parameters (frequency 1 MHz, intensity 1–3 W/cm^2^, microbubble-to-cell ratio 20:1–70:1, exposure time 1–2 min, duty cycle 20–100%) and cell viability was determined. Viability was lowered with increasing ultrasound intensity, duty cycle and microbubble concentration. Furthermore, the authors demonstrated that not all delivery vectors had increased transfection efficiency when combined with USMB. PEI/pDNA + USMB led to the highest transfection efficiency compared with the control, followed by rAAV + USMB. Transfection efficiency using L/siRNA was not enhanced by USMB compared with the control.

The intracellular enhancement of uptake of fluorescently labelled siRNA encapsulated in mPEG-PLGA-PLL nanoparticles by rat RPE cells was investigated by Du et al. [90]. The authors observed a 1.5-fold increase in nanoparticle uptake (48.39%) when cells were treated with ultrasound alone (1 MHz frequency, 0.5 W/cm^2^ intensity, 60 s exposure time) compared with the untreated control group (32.17%). Interestingly, nanoparticle uptake did not differ significantly between the cells treated with ultrasound and USMB groups, which was attributed to possible damage in the microstructure of RPE cells (microvilli or caveolae) induced during microbubble cavitation. In 2017, the same research group conducted an in vivo animal study to investigate the transfection efficiency of the same nanoparticles loading PDGF-BB siRNA [91]. In contrast to their in vitro uptake study, higher values of ultrasound intensity and exposure time were used (intensity 2 W/cm^2^, exposure time 5 min). In vivo results indicated an enhanced nanoparticle uptake when combined with USMB (transfection efficiency 18.22%), which was significantly greater than eyes treated with nanoparticles + ultrasound (10.67%) or nanoparticles alone (3.74%). Histological examination of enucleated eyes showed that retinal layers were well preserved without any damage in the photoreceptor layer or inflammation.

In conclusion, the above studies have demonstrated that USMB can induce a reversable disruption of the BRB, enhance the extravasation of molecules to the neural retina, and improve the intracellular uptake of macromolecules, genes and nanoparticles in retinal cells. The chosen ultrasound parameters are crucial for the efficacy, as well as the safety of the treatment. As seen in the following sections, this is a common observation among all USMB applications.

### 4.2. Glaucoma

Glaucoma refers to a group of eye neuropathies characterized by retinal ganglion cell (RGC) damage and apoptosis. One of the clinical hallmarks of glaucoma is optic disc excavation (Figure 4C). It is the second leading cause of blindness worldwide (following cataract), with the most common type being primary open-angle glaucoma. Multiple factors are associated with the development of glaucoma, such as age, family history, diabetes, treatment with steroids and hypertension [92,93]. Reducing intraocular pressure (IOP) is the most common approach for the prevention of glaucoma progression, which is typically performed via topical administration of eye drops (prostaglandin analogues, beta blockers, alpha 2-adrenergic agonists, carbonic anhydrase inhibitors) [94,95,96,97]. Topical administration of eye drops is characterized by low bioavailability due to the rapid elimination of eye drop solutions and systemic drug absorption through blood vessels in the conjunctiva [58]. In addition, this treatment method is associated with compliance rates of approximately 55% of patients, in chronic treatment of glaucoma, related to patient-perceived problems such as forgetting doses [98]. Other treatment methods are surgical interventions such as cyclo-cryocoagulation, laser treatment (laser trabeculoplasty and cyclophotocoagulation), filtration and non-filtration surgeries [99]. Despite the efficacy of these treatment methods to reduce IOP, the need to treat RGCs and optic nerve degeneration is still unmet.

A number of studies focused on the USMB-mediated treatment of glaucoma by enhancing the delivery of neuron growth factors and genes that can prevent RGC apoptosis and enhance optic nerve protection. Shen et al. investigated the uptake of mouse neuron growth factor (mNGF) by RGCs in a rabbit model for intraocular hypertension [100]. They have shown that USMB (1 MHz frequency, 0.5 W/cm^2^ intensity, 1 min exposure time) in combination with mNGF significantly improved the flash visual evoked potential (F-VEP) scores (F-VEP assesses the function of optic nerve myelin and axons by measuring the electric potential generated upon application of a light stimulus). In addition, retinal thickness was significantly increased compared with controls; photoreceptors had normal appearance without degeneration, and RGCs were normal in structure both in the retina and optic nerve.

The potential of USMB-mediated gene transfection of RGCs was investigated in vitro and in vivo [101,102]. Li et al. investigated the delivery of the bcl-xl gene (i.e., the main gene from the bcl-2 family expressed in the rat retina, known for its anti-apoptotic activity) to RGCs and its role in the prevention of cell apoptosis [101]. Non-transfected RGCs were shrunken, rounded and detached, showing extensive apoptosis. On the other hand, USMB-combined delivery of bc1-xl reduced the number of apoptotic cells, showing that USMB could partially prevent RGC death. Gene delivery of the rAAV-EGFP gene to RGCs was investigated in a study by Xie et al. [102]. USMB treatment was performed after intravitreal injection of the gene with or without lipid microbubbles (ultrasound frequency 0.3 MHz, intensity 0.5 W/cm^2^, exposure time 1 min). Examination of retinas 28 days after treatment revealed that the highest RGCs transduction rate was observed in the eyes that received combined gene delivery and USMB (rAAV-EGFP 12.75%, rAAV-EGFP + ultrasound 15.78%, rAAV-EGFP + USMB 19.48%).

A different application of USMB in the treatment of glaucoma was demonstrated by Yamashita et al. by showing that the delivery of genes to the anterior eye could improve and accelerate wound healing following one of the invasive methods used for the treatment of glaucoma, such as trabeculoplasty [103]. First, the transfection efficiency of pDNA (pEGFP-N2 gene) in combination with ultrasound bubble liposomes (USBL) was investigated in vitro in corneal epithelial cells. Cells were treated at 1 MHz ultrasound frequency and exposure time of 20 sec. Ultrasound intensity varied between 0.8 and 1.2 W/cm^2^. The highest number of GFP-positive cells was found at the highest ultrasound intensity, with no significant changes in cell viability. Next, transfection efficiency was compared between bubble liposomes (BLs) and conventional microbubbles (Optison^TM^, GE Healthcare), revealing that the ratio of GFP-positive cells over the total amount of cells treated with USBL was approximately 2 times higher than for the cells treated with USMB. Gene expression in rat eyes was then compared between USBL- and USMB-mediated delivery. In vivo results were in agreement with the in vitro study, indicating that plasmid + USBL resulted in a higher number of GFP-positive cells compared with plasmid alone, plasmid + ultrasound and plasmid + USMB. USBL-mediated GFP-positive cells were mostly located beneath the conjunctival epithelium in the area that was treated with ultrasound and GFP was found localized in the cytosol (Figure 5D). The structure of the conjunctiva was well preserved, and no hemorrhage or edema was found.

In conclusion, the studies discussed above have shown the potency of USMB to enhance the delivery of a neuron growth factor and various genes and enhance the protection of RGCs in glaucoma.

### 4.3. Diabetic Retinopathy

Diabetic retinopathy (DR) is a disease that occurs in almost all patients with type 1 diabetes and more than 60% of patients with type 2 diabetes. It can be classified into (i) non-proliferating, when microaneurysms and hemorrhages appear in the retina and (ii) proliferating, when new blood vessels (retinal neovascularization) appear on the surface of the retina or optic disc. These abnormal blood vessels may bleed, leading to vitreous hemorrhage, fibrosis and retinal detachment. DR is frequently associated with diabetic macular edema, characterized by the deposition of hard exudates at the central retina, increased vascular permeability and microaneurysms (Figure 4D) [104,105,106]. DR has been traditionally treated with focal laser photocoagulation, surgical vitrectomy or with administration of corticosteroids with anti-inflammatory and antiangiogenic action (e.g., triamcinolone acetonide). The latter can be administered as intravitreal injections or retinal implants [107]. Following the improved therapeutic results in the treatment of AMD, the anti-VEGF drugs pegaptanib, ranibizumab and bevacizumab are also used in the treatment of DR and diabetic macular edema patients with positive results [105,108,109].

One approach where USMB is used against DR is to enhance the delivery of endostatin (ES). ES is a fragment antibody (M_W_ 20 kDa) known for its inhibitory function in endothelial proliferation, anti-angiogenetic and antitumoral growth action [110]. Xu et al. developed cationic microbubbles (CMB) and studied the delivery ES-GFP plasmid to human retinal vascular endothelial cells, aiming to treat DR with inhibiting angiogenesis [111]. The total amount of ES protein was determined in cells, 48 h post-transfection (frequency 1 MHz, intensity 1 W/cm^2^, exposure time 1 min). ES protein level was significantly elevated in cells transfected with CMB + ultrasound, compared to cells treated with neutral nanobubbles (NMB) + ultrasound, or liposomes.

Another approach for the treatment of angiogenic eye diseases such as DR was followed by Kowalczuk et al., who investigated the delivery of genes in the ciliary muscle [112]. As this muscle is located between the anterior and posterior eye segments, it was hypothesized that its transfection may allow for the production of proteins with anti-angiogenic or anti-inflammatory action. Microbubbles were mixed with pDNA encoding for Gaussia luciferase (pCMV-Gluc-1) or GFP (pEGFP-C1) and were injected into the ciliary muscle of rat eyes. Sonications were performed at 1 MHz frequency, 2 W/cm^2^ intensity (0.7 MPa PNP), and 2 min exposure time. Analysis of aqueous and vitreous humors samples collected 7 days after treatment showed that luciferase secretion in the pCMV-Gluc-1 + USMB group was 2.6-fold higher compared with the group treated with pCMV-Gluc-1 alone. Enhanced expression of GFP in ciliary muscle cells and around the ciliary body was seen 7 days after treatment with pEGFP-C1 + USMB (Figure 5E). No tissue damage was detected in the ciliary muscle, cornea or retina, showing that USMB treatment was safe at the specific settings. Temperature elevations were monitored in the lens and ciliary muscle during ultrasound exposure. A temperature increase of 3.7 °C and 7.3 °C was seen in the lens and ciliary muscle, respectively, but normal temperature was recovered a few seconds after treatment was completed. No alterations in the transparency of the lens were observed for up to a month later.

In conclusion, there is much less literature compared to AMD and glaucoma, though there are also therapeutic opportunities for USMB in DR.

### 4.4. Proliferative Vitreoretinopathy

Proliferative vitreoretinopathy (PVR) is an abnormal wound-healing process as a result of surgical failure in the treatment of rhegmatogenous retinal detachment. PVR is characterized by fixed retinal folds (Figure 4E), and proliferation and migration of RPE and RGC cells that result in cell collections on the retinal surface and vitreous cavity [113,114]. A pool of growth factors, such as transforming growth factor (TGF-β2), platelet-derived growth factor (PDGF), and hepatocyte growth factor, are known contributors to the progression of PVR [115,116,117]. PVR is primarily treated with surgical intervention for vitrectomy and membrane peeling [118].

Zheng et al. investigated the role of USMB in the enhancement of rAAV-siRNA delivery to inhibit the expression of two of the growth factors (TGF-β2 and PDGF-B) associated with proliferation of PVR [119]. The study was conducted using a disease animal model for PVR. Three days after induction of PVR, animals were treated with TGF-b2-siRNA, or PDGF-B-siRNA, or a combination of the two siRNAs. In addition, one group received the two siRNAs combined with USMB (frequency 1 MHz, intensity 2 W/cm^2^, exposure time 300 sec). Disease progression (proliferation level) was classified on a scale of 0–4, based on the morphological characteristics in the fundus photographs (vitreous haze, retinal folds and epiretinal membrane formation). Fourteen days after treatment, the proliferation level for eyes that received the combination of siRNAs (with and without USMB) was significantly lower than the control groups and the eyes treated with one of the two siRNAs, indicating that combined treatment with USMB decelerated proliferation. On the contrary, 28 days post-treatment, significantly lower proliferation grade was seen in the eyes that received combination of siRNAs + USMB compared with all other treatment groups, with 20% of the eyes in the USMB group showing no further proliferation between days 14 and 28. Twenty-eight days after treatment, the extent of retinal detachment, the number of effector cells (RPE, RGC, fibroblasts and macrophages) and proliferative membrane formation were dramatically reduced in the USMB-treated eyes compared to all other groups. Protein expression levels of TGF-β2 and PDGF-B in the USMB-treated group were only half of the untreated group (639.85 vs. 1363.15 pg/mL for TGF-β2, and 66.94 vs. 137.76 pg/mL for PDGF-B), confirming that combined treatment of siRNAs + USMB can limit the expression of TGF-β2 and PDGF-B, and eventually, delay the progression of PVR.

### 4.5. Retinitis Pigmentosa

Retinitis pigmentosa (RP) is a group of hereditary ocular diseases caused by degeneration of rod and cone photoreceptor and RPE cells in the retina. At early RP stages during adolescence, patients experience difficulties with vision adaptation in darkness and night brightness (due to degeneration of rod photoreceptors), while in young adulthood, mid-peripheral vision is lost, followed by loss of central vision by the age of 60 years (due to degeneration of cone photoreceptors). Typical clinical signs are peripheric retina bone-spicule deposits (Figure 4F). At the progressed disease stages, degeneration of cells in the INL (amacrine, bipolar and horizontal neuron cells) and RGC might occur [120,121]. Currently, no effective treatments are available for RP, though vitamin therapy can be used for the preservation of photoreceptor function. Nutritional supplements aim to provide protection to retinal cells from oxidative damage, optimize key elements of photoreceptor structure, and maintain the integrity of retinal blood oxygenation network (i.e., retinal blood vessels and choroid). Vitamin A is especially important, as it plays a role in the formation of rhodopsin, a receptor protein with critical function in rod photoreceptors. Supplementation with oral vitamin A is the most widely followed method for slowing photoreceptor degeneration in RP patients [120,122,123].

As previously discussed in the context of the treatment of wet AMD, numerous studies have demonstrated the USMB-mediated enhanced intracellular uptake of macromolecules [82], genes [83,84,85,86,87,88,89] and nanoparticles [90,91] in the neural retina and RPE. A similar USMB approach could also be feasible for treating RP.

### 4.6. Retinoblastoma

Retinoblastoma (RB) (Figure 4G) is an uncommon pediatric cancer, yet it is the primary intraocular malignancy in children. It is caused by a mutation in the RB1 gene and is fatal if untreated [124,125]. Replacement of traditional treatments such as eye enucleation and radiotherapy with chemotherapy has increased the survival rate to nearly 100% [126,127,128,129]. The treatment protocol to be used for RB therapy depends on laterality (tumor diagnosis in one or both eyes) and the International Classification of Retinoblastoma (ICRB) stage. In bilateral tumors, the presence of germline disease, or if optic nerve or choroid invasion is suspected, patients receive intravenous chemotherapy (IVC) to prevent metastases. ICRB classifies unilateral tumors as five subgroups based on tumor size, location and the presence of seeds. Unilateral tumors are treated with cryotherapy or transpupillary thermotherapy if they are small (≤3 mm), or larger than 3 mm and in the vicinity of macula and optic nerve. Extensive unilateral tumors and tumors with seeds are treated with intra-arterial chemotherapy (IAC). Usually, systemic toxicity is mild, and no ophthalmic toxicities have been reported as a result of IVC. IAC is more efficacious compared with IVC as the drug dose reaching the eye is increased by 10-fold [130,131]. This results in reduced duration of treatment and systemic toxicity. Despite the tremendous improvement in RB treatment with IVC and IAC, chemoresistant and recurrent tumors do occur often, caused by inadequate drug delivery into the vitreous or subretinal seeds [130,132].

The role of USMB in the enhancement of intracellular uptake of the chemotherapeutic agent doxorubicin (dox) in RB was studied in vitro in human RB cells [133]. First, the effect of USMB on cell viability was tested using various ultrasound intensities (0.3–1.0 W/cm^2^), at frequency of 1 MHz and exposure time of 10 sec, in absence of the drug. Maximum cell viability (97.9% of the untreated cells) was obtained at the lowest intensity. Next, the viability of RB cells after combined treatment with dox and USMB (at 0.3 W/cm^2^) was studied for 3 days. On the first day after treatment, no significant changes between treated and untreated cells were detected. However, 2 days post-treatment, cells exposed to dox + USMB had significantly reduced viability (34.9%) compared with cells treated with dox only (50.9%). Considering the mechanism of action of dox (i.e., intercalation into DNA and damage to cellular membranes, DNA, and proteins by generation of free radicals), cell apoptosis is not immediately seen after treatment. This might explain why the first induction of cell apoptosis was seen not earlier than 2 days post-treatment.

### 4.7. Eyelid Malignant Melanoma

Eyelid melanoma is an uncommon cutaneous malignancy that results from malignant proliferation of melanocytes in the epidermis of the eyelid skin (Figure 4H) [134,135]. Despite being generally similar to melanomas encountered anywhere on the skin in the head and neck area, eyelid melanoma is associated with sentinel lymph node metastasis due to the unique lymphatic drainage of the periocular skin [136]. Depending on the disease stage, current treatment methods include tumor excision, lymph node dissection and radiotherapy. Although these treatment methods are satisfactory, they are often associated with cosmetic issues.

Considering their superficial location, USMB-mediated treatment of eyelid malignancy could be a non-invasive alternative to surgical excision of the tumor. Sonoda et al. studied the uptake of a chemotherapeutic agent (bleomycin) enhanced by USMB in eyelid malignant melanoma in vitro and in vivo [137]. Mouse melanoma cells were treated with USMB (1 MHz frequency, 1 W/cm^2^ intensity, 60 s exposure time) in combination with bleomycin (50 nm–5 μM concentration). A significant reduction in viability was observed only at the two highest drug concentrations in the cells treated with bleomycin + ultrasound. On the other hand, viability dropped at all bleomycin concentrations in the USMB treated cells, which was more dramatic with increasing drug concentration.

In the in vivo study, bleomycin and microbubbles were injected intratumorally and treatment efficacy was assessed by calculating the mean relative tumor weight for up to 8 days after treatment [137]. An ultrasound intensity of 2 W/cm^2^ and exposure time of 240 s were used. Tumors treated with 0.125 mg/mL bleomycin + USMB decreased in weight 2 days after treatment and continued shrinking until the last measurement. For the same drug concentration and in combination with ultrasound only, tumors grew in size over time and, on the last measurement, the average relative weight had increased by three times. Tumors in the control group were treated with bleomycin only at concentrations 0.25–2 mg/mL, and though these concentrations were higher than those used in the ultrasound and USMB groups, tumor growth was decelerated, but no reduction in size was observed. Furthermore, in the bleomycin + USMB group, for the rest of the drug concentrations tested (0.06 mg/mL, 0.25 mg/mL and 0.5 mg/mL), tumors initially increased in weight (day 2) but later continuously decreased in weight until day 8. Histological assessment of tumor samples revealed that in tumors treated with chemotherapy only, melanoma cells were actively dividing, and cell nuclei were pigmented and heterogeneous. In contrast, in the cases where tumor weight reduction was seen, a small amount of melanoma cells were present in the deepest part of the tumor, while the rest of the tumor area was occupied by necrotic cells. No histologic abnormalities were seen in the tumor peripheral tissue. Consequently, Sonoda et al. clearly showed that addition of microbubbles led to a significant decrease in tumor size at all drug concentrations and could enhance treatment efficacy using lower concentrations than the non-USMB treatment groups.

### 4.8. Corneal Opacity

Corneal opacity is the medical condition occurring when corneal clarity is lost. It appears as loss of transparency of the corneal membrane (Figure 4I), which affects the transmission and scattering of light, resulting in reduced visual acuity. Factors that can cause corneal opacity include corneal infection, injury, and edema [138,139]. Depending on the etiology, corneal opacity is treated with antibiotics, corticosteroids, antiviral drugs, or other interventions such as phototherapeutic keratectomy, or in severe cases, with keratoplasty (corneal transplantation) [140,141,142,143,144]. Permeability of cornea after topical administration of drug compounds largely depends on the drug molecule’s solubility and polarity: the corneal epithelium and endothelium have a strong lipophilic character and resist penetration of polar molecules, while the corneal stroma is hydrophilic and resists penetration of non-polar molecules [145,146].

USMB-mediated transfection efficiency of pDNA (pEGFP-N2 gene) in the cornea was investigated using rabbit corneal epithelial cells in vitro and in rabbit cornea in vivo [147]. In the in vitro study, cells were exposed to 1 MHz frequency, 15–120 s exposure time and 0.5–2.0 W/cm^2^ ultrasound intensity. An increase in the percentage of GFP-positive cells was seen at 60 and 120 s exposure times, without a significant reduction in cell viability. Intensity of 0.5 W/cm^2^ resulted in the lowest number of GFP-positive cells, and any increase higher than 1.5 W/cm^2^ induced cell toxicity. Rabbit eyes that received plasmid alone or pDNA + ultrasound had mild GFP expression on the corneal stroma. In contrast, eyes that received pDNA + USMB (intensity 2 W/cm^2^, intra-corneal injection of microbubbles) showed the highest amount of GFP-positive cell expression, colocalized with the treated area (Figure 5F). It is hypothesized by the authors that because corneal composition consists of multiple cell layers and extracellular matrix, higher ultrasound intensities are required to induce microbubble cavitation bioeffects in vivo than in vitro, where a single layer of cells is exposed to ultrasound. In vivo, ultrasound intensity higher than 3 W/cm^2^ caused corneal haziness, which was resolved later without treatment. In the pDNA + USMB group (2 W/cm^2^ intensity for 120 s exposure time), GFP-positive cells were detected 1 day after treatment and the highest density was measured 4 days and 8 days after treatment. On day 14 post-treatment, GFP-cell density was significantly lower, and was no longer detected on day 30. Microscopic examination of corneal sections treated with USMB revealed that GFP-positive cells were located inside the corneal stroma, and no GFP-positive cells were detected in untreated corneal tissue, ciliary epithelial cells, trabecular meshwork, lens epithelial cells or retina. Light and electron microscopy performed 48 h post-treatment revealed no corneal damage. Consequently, this study showed that USMB can enhance gene delivery in the anterior eye without inducing any adverse effects.

**Table 1:** Summary of the studies investigating the use of USMB in the treatment of ocular diseases.

Study Model	Delivered Compound	Microbubbles and In Vivo Administration Site	Ultrasound Parameters	Efficacy	Safety	Reference
In vivo, (rabbit)	Fluorescein	Definity^®^, intravenous	2 MHz frequency, 0.2 and 1.7 MI, 5 min exposure time	Alteration in the diameter of uveal blood vessels observed in 20% and 80% of eyes treated al low and high MI, respectively. At high MI, vasoconstriction and extravasation of fluorescein were observed, and the mean number of altered segments in blood vessels was higher than at low MI.	No bleeding.	[79]
In vivo, (rat)	Gd	Definity^®^, intravenous	0.69 MHz frequency, 0.81, 0.88 and 1.10 MPa PNP (MI 0.98, 1.06, and 1.32, respectively), 60 s exposure time.	Immediate increase in Gd signal after treatment indicating BRB disruption. For the two lower pressures, Gd signal was lowered 3.5 h post-treatment, revealing reversibility of BRB disruption, but not at 1.10 MPa PNP.	Extravasated erythrocytes in the nuclear layers of the retina with more severe damage at 1.10 MPa.	[80]
In vivo, (rat, mouse)	Evans blue, IgG, IgM	Definity^®^, intravenous	1.1 MHz frequency, 0.36–0.84 MPa PNP (MI 0.34–0.80), 120 s exposure time.	Extravasation of Evans blue, IgG and IgM was observed in neural retina (INL and RGC) suggesting that the vascular plexi within these layers were permeabilized. No evidence for molecule transfer across the choroid and into the RPE.	Evidence for morphological damage, reactive gliosis, neuroinflammation and presence of erythroid cells. No megakaryocyte infiltration.	[81]
In vitro (RPE, Müller glia, photoreceptors)	IgG	Custom-made NBs with shells made of DPPC/DSPE-PEG(2k)-Ome and PFP inner gaseous phase	1 MHz frequency, 0.5 W/cm^2^ intensity, 30 s exposure time	Increase in the intracellular uptake of IgG after treatment with USNB was cell line-dependent. USNB efficacy is highly dependent on ultrasound intensity and exposure time.	N/A	[82]
In vitro (RPE), in vivo (rat)	PEI/pEGFP	SonoVue^TM^, subretinal injection	1 MHz frequency, 1–3 W/cm^2^ intensity, 1–5 min exposure time	In vitro: higher exposure time resulted in higher number of GFP-positive cells and decreased cell viability. In vivo: high density EGFP-positive cells were observed in animals treated with PEI/pEGFP + USMB, predominantly distributed in the retina.	No tissue damage.	[83]
In vivo (rat)	pEGFP-N1	SonoVue^TM^, subretinal injection	1 MHz frequency, 2 W/cm^2^ intensity, 5 min exposure time	The highest EGFP-positive signal was observed in the PEI/pDNA + USMB group, distributed in neural retina and RPE cells. The same trend was observed in the quantification of EGFP gene copy number and the EGFP mRNA expression level in the RPE and neural retina.	No evidence for corneal and retinal tissue damage, no morphological alterations and no inflammatory cell infiltration.	[84]
In vivo (rabbit)	pEGFP-N2	Custom-made BL. Shells made by DSPC/DSPE-PEG (2k)-Ome, inner phase PFP gas. Intravitreal injection	3 MHz frequency, 0.15 W/cm^2^ intensity, 60 s exposure time	Highest amount of GFP-score in the plasmid and USBL group. GFP-positive cells were colocalized with the areas exposed to ultrasound and were detected in the ONL.	No obvious tissue damage.	[85]
In vitro (human retinal pigment epithelium cells), in vivo (rat)	rAAV-EGFP	SonoVue^TM^, subretinal injection	1 MHz frequency, 0.5–2 W/cm^2^ intensity, 1–5 min exposure time	In vitro, combined treatment with USMB resulted in the highest transduction efficiency than treatment with ultrasound only. In vivo, quantification of EGFP signal revealed significantly elevated values for the USMB group on the first 35 days post-treatment. EGFP-positive cells USMB group were found in neural retina and RPE cells.	No evidence for tissue damage.	[86]
In vitro (rat RPE cells), in vivo (rat)	rAAV2-EGFP	SonoVue^TM^, subretinal injection	1 MHz frequency, 0.2–3 W/cm^2^ intensity, 15–300 s exposure time	Compared to the control group either ultrasound or microbubbles alone, but not their combination, increased rAAV-EGFP transduction of RPE-J cells in vitro. USMB-enhanced treatment resulted in a higher expression of EGFP in vivo. An increase in GFP-fluorescence was found until day 35 and reduced up to 120 days post-treatment. GFP signal was found in RPE and neural retina.	Adverse effects in cell viability in vitro observed at intensity of 3 W/cm^2^. In vivo, all retina cell layers were well preserved without photoreceptor loss or inflammation.	[87]
In vivo (rat)	Lipofectamine-formulated fluorescently labelled-siRNA	SonoVue^TM^, intravitreal injection	1 MHz frequency, 2 W/cm^2^ intensity, 300 s exposure time	The greatest quantity of transduced cells was observed in the group treated with lipofectamine-formulated siRNA combined with USMB. No fluorescence was detected in either the untreated or treated with naked siRNA + ultrasound groups.	No significant cell viability reduction observed 12 h after transfection. Retina cell layers were well preserved without photoreceptor loss, nuclear layer vacuolation, or inflammation.	[88]
In vitro (human RPE cells)	rAAV-EGFP, PEI/pDNA and L/siRNA	SonoVue^TM^	1 MHz frequency, 1–3 W/cm^2^ intensity, 60–120 s exposure time	Transfection efficiency of rAAV and PEI/pDNA vectors significantly improved when gene delivery was combined with USMB, in contrast to the L/siRNA efficiency that was benefited by ultrasound alone. Combined treatment with USMB did not cause structural alterations on the pDNA.	N/A	[89]
In vitro (rat RPE cells)	Fluorescently labelled siRNA encapsulated in mPEG-PLGA-PLL nanoparticles	SonoVue^TM^	1 MHz frequency, 0.5–2 W/cm^2^ intensity, 30–60 s exposure time	Highest nanoparticle uptake observed in cells treated with ultrasound alone. Combination with USMB did not improve the nanoparticle uptake.	At the settings with the highest nanoparticle uptake, a temperature increase of 1.9 °C was reported with no influence on cell viability.	[90]
In vivo (rat)	Fluorescently labelled PDGF-BB siRNA encapsulated in mPEG-PLGA-PLL nanoparticles	SonoVue^TM^, intravitreal injection	1 MHz frequency, 2 W/cm^2^ intensity, 5 min exposure time	The highest transfection efficiency in neural retina was achieved after combined treatment with USMB.	No evidence for tissue damage observed. All layers of the retina were well preserved without photoreceptor loss or inflammation.	[91]
In vivo (rabbit, intraocular hypertension animal model)	mNGF	SonoVue^TM^, intravitreal	1 MHz frequency, 0.5 W/cm^2^ ultrasound intensity, 60 s exposure time	Function of optic nerve myelin and axons was improved in the group that received mNGF + USMB. Retinas treated with mNGF + USMB had clear and orderly arranged cell layers. The thickness of the inner and outer plexiform layers was nearly normal. Rod and cone cells were normally aligned without degeneration, and RGC were normal in structure.	N/A	[100]
In vitro (rat RGC)	pEGFP-N1 and bcl-xl	SonoVue^TM^	0.3 MHz frequency, 0.25–1.25 W/cm^2^ intensity, 30–120 s exposure time	Improved transfection efficiency observed in pEGFP-N1 + USMB group. USMB-mediated bc1-xl transfection had a role in protection of RGCs from apoptosis, but not in complete apoptosis prevention.	N/A	[101]
In vivo (rat)	rAAV2-EGFP	Custom-made lipid microbubbles (shell: DSPC, 1,2-DSPE, DSPA, inner gas: PFP), intravitreal injection	0.3 MHz frequency, 0.5–2.5 W/cm^2^ intensity, 60 s exposure time	Greatest EGFP expression was observed in retinas treated with rAAV2-EGFP + USMB. The majority of GFP-positive cells were RGC.	No structural, morphological alterations, no cellular infiltration in the vitreal cavity.	[102]
In vitro (rabbit cornea epithelial cells), in vivo (rat)	pEGFP-N2	Custom-made BL. Shells made by DSPC/DSPE-PEG (2k)-Ome, inner gaseous phase PFP gas. Subconjunctival injection.	1 MHz frequency, 0.8–1.2 W/cm^2^ intensity, 20–60 s exposure time	In vitro: The ratio of GFP-positive cells treated with USBL was about 2 times higher than the USMB group. No observed decrease in cell viability in any of the experimental groups. In vivo: GFP-positive cell density in eyes treated with USBL was significantly higher than the groups that received plasmid only, plasmid + ultrasound and plasmid + USMB. GFP-positive cells were mostly located beneath the conjunctival epithelium of the area exposed to ultrasound. No significant number of GFP-positive cells was observed in any other part of the eye.	The structure of conjunctiva was well preserved. No signs of hemorrhage, edema or inflammation.	[103]
In vitro (human retinal vascular endothelial cells)	ES-GFP	NMB: DPPC/DSPE-PEG2000 and cationic microbubbles CMB: DPPC/DSPE-PEG2000-Biotin/DC-Chol, containing PFP gas	1 MHz frequency, 1 W/cm^2^ intensity, 1 min exposure time	CMBs had higher plasmid binding compared with NMBs. In cells treated with CMBs, the level of VEGF, Bcl-2, and Bcl-xl mRNA was decreased.	N/A	[111]
In vivo (rat)	pCMV-Gluc-1, pVAX1-LacZ, pEGFP-C1	Artison, intra-muscle injection (ciliary muscle)	1 MHz frequency, 0.7 MPa PNP, 120 s exposure time	One week after treatment, the group treated with pCMV-Gluc-1 plasmid + USMB had the greatest expression of luciferase. Enhanced expression of β-galactosidase in the ciliary muscle cells and sporadically around the ciliary body was observed microscopically. Similar enhancement and localization site of GFP protein observed in the pEGFP-C1 + USMB group.	No apoptotic cells detected in the conjunctiva, retina or cornea. A temperature increase of 3.7 °C in the lens and 7.3 °C in the ciliary muscle measured during ultrasound exposure. Normal temperature was immediately recovered. No alterations in the transparency of the lens for up to a month post-treatment.	[112]
In vivo (rat, proliferative vitreoretinopathy disease model)	rAAV2-TGF-β2-siRNA and rAAV2-PDGF-B-siRNA	SonoVue^TM^, intravitreal injection	1 MHz frequency, 300 s exposure time	In the group treated with siRNAs + USMB, retinal morphologic alterations progressed slower than control groups. The numbers of effector cells, such as RPE cells, glial cells, fibroblasts and macrophages, and the incidence of retinal detachment, and proliferative membrane formation were significantly less than the eyes treated without USMB.	N/A	[119]
In vitro (human RB cells)	Doxorubicin	Artison	1 MHz frequency, 0.3–10 W/cm^2^ intensity, 10 s exposure time	No significant differences in cell viability observed 24 h post-treatment between cells treated with doxorubicin alone and doxorubicin + USMB. Viability of cells exposed to doxorubicin + USMB was significantly lower compared with cells exposed to doxorubicin alone 48 and 72 h, but not 24 h post-treatment.	N/A	[133]
In vitro (mouse melanoma cells), in vivo (mouse)	Bleomycin	Optison^TM^, intratumoral injection	1 MHz frequency, 1–2 W/cm^2^ intensity, 60–240 s exposure time	Combination of bleomycin and USMB in vitro resulted in a significant decrease in cell viability at all concentrations tested. In vivo, in the bleomycin + USMB group, for drug concentrations of 0.06 mg/mL, 0.25 mg/mL and 0.5 mg/mL, tumors initially increased in weight but later had a continuous decrease until day 8. Tumors treated with 0.125 mg/mL bleomycin + USMB responded immediately after the 1st treatment with a continuous reduction in size. No reduction in size was observed in the group treated with bleomycin alone.	In vivo, temperature inside the tumor increased from 34 to 37 °C. The temperature of ultrasound probe changed in similar manner. No histological abnormalities were seen in the brain, lung, liver and heart.	[137]
In vitro, (rabbit corneal epithelial cells), in vivo (rabbit)	pEGFP-N2	Optison^TM^, intracorneal	1 MHz frequency, 0.5–2 W/cm^2^ intensity, 15–120 s exposure time	In vitro: The greatest amount of GFP-positive cells ratio was significantly greater in samples treated with USMB. In vivo: The eyes that received plasmid + USMB showed the highest number of GFP-positive cells. GFP-positive cells appeared one day after treatment. Fluorescence intensity increased the first 8 days, significantly decreased on day 14, and was not measurable on day 30 after treatment. GFP was mainly located inside the corneal stroma.	Immediate corneal stroma haziness appeared at intensity >3 W/cm^2^, which spontaneously resolved immediately after treatment. No corneal damage, such as opacity or persistent epithelial defects, was observed.	[147]

## 5. Safety and Tolerability of USMB in Ocular Therapeutic Applications

**Figure 6:**
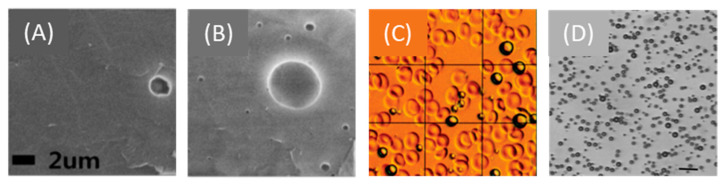
Commercially available microbubbles. Cryo-SEM images of (**A**) SonoVue^TM^ and (**B**) Definity^®^ microbubbles. Adapted from [158], MDPI, 2013. (**C**) Optison^TM^ microbubbles against a background of red blood cells. Adapted with permission from [159], RSNA, 2010. (**D**) Optical microscopy image of Sonazoid^TM^ microbubbles. Adapted with permission from [160], Elsevier, 2008.

The safety of USMB is generally reliant on the individual components that play a role during treatment: the microbubbles and the ultrasound settings. The presently commercially available microbubbles (Figure 6) are generally well tolerated by patients [149]. Currently, there are four microbubble products approved for use in ultrasound imaging; these are OptisonTM (GE Healthcare, Little Chalfont, UK), SonoVueTM (Bracco, Milan, Italy), Definity^®^ (Lantheus Medical Imaging, North Billerica, MA, USA) and Sonazoid^TM^ (GE Healthcare). Microbubbles are well tolerated, though there are some contraindications related to the microbubble shell-forming agent. For the phospholipid-containing agents (SonoVue^TM^, Definity^®^ and Sonazoid^TM^), a contraindication exists for patients who have a history of hypersensitivity to phospholipids (manifesting, for instance, as an egg allergy). SonoVue^TM^, Definity^®^ and Sonazoid^TM^ contain polyethylene glycol (PEG) as an excipient, as it is present in the microbubble shell. PEG reduces opsonization and interaction with cells, and increases formulation stability [150]. Recently, some case reports were published after anaphylactic reactions to PEG components, indicating the potential for immunoglobulin E-mediated type I hypersensitivity reactions. [151,152]. The FDA MedWatch alert identified 11 anaphylaxis cases, including 2 deaths, over a period of several decades. In 2021, the American Society of Echocardiography published an expert consensus statement with recommendations for laboratory policies on microbubbles containing PEG, stating that SonoVue^TM^ and Definity^®^ are contraindicated in patients with known hypersensitivity to these agents or their components, but also contraindicated in patients with known hypersensitivity to PEG (Sonazoid^TM^ is currently not approved for use in the United States) [153]. The FDA states that Optison^TM^ is an alternative to Definity^®^ and SonoVue^TM^ in patients with hypersensitivity to PEG, as it contains albumin rather PEG [154]. Additionally, a recent animal study on the pharmacokinetics of PEGylated microbubble administration indicated that microbubble blood clearance can be accelerated upon repeated administration. This reduction in microbubble half-life is due to opsonization by IgM and IgG anti-PEG antibodies that are produced by the immune system after the initial microbubble administration [155]. Investigation into the clinical relevance of this effect for microbubbles has yet to be investigated.

Patients with a hypersensitivity to blood products, such as human albumin, are advised against treatment with Optison^TM^ microbubble products. Another parameter in which the current microbubbles can be categorized is the gas used as disperse phase. For Definity^®^ and Optison^TM^, perflutren gas is used, whereas for Sonazoid^TM^ and SonoVue^TM^, perfluorobutane and sulphur hexafluoride are used, respectively. For all microbubble products, the contained gasses are usually eliminated quickly from the bloodstream, typically without any adverse effect, unless the patient is hypersensitive to the specific gas used [6,156,157].

The induced bioeffects are highly related to the type of cavitation (stable or inertial) microbubbles undergo during USMB treatment. Commercial microbubbles are polydisperse, i.e., there is variation in size within the microbubble population [6,161]. During exposure to ultrasound, microbubbles with different sizes oscillate in a non-uniform manner. Specifically, while some microbubbles with a certain size oscillate in a safe manner, bubbles with different size might simultaneously induce unwanted side effects. As a result, polydispersity is related to a broad spectrum of bioeffects, which makes efficacy and safety more difficult to control. Recently, in order to rectify this safety issue, development of monodisperse microbubbles for imaging and therapy has been gaining momentum [6,31,162].

Prior to clinical translation, a method that will allow the monitoring of the intensity of induced bioeffects in real time is needed. It has been previously reported that acoustic microbubble emissions can be used to monitor the presence, the type, the location and the level of cavitation in the brain, in real-time [163]. The harmonic, subharmonic and ultraharmonic components of microbubble signals can be recorded by a cavitation detector and, using a feedback control algorithm, the acoustic pressure generated can be adjusted [43,164,165]. Touahri et al. [81] used the same method as O’Reilly and Hynynen [165] to verify the effect of focused ultrasound on microbubbles when sonicating rat retinas, by monitoring the generation of subharmonic signals. Despite this monitoring, some evidence for neuroinflammation and the presence of erythroid cells was found in the areas where the BRB was disrupted. In the future, feedback control methods specifically designed to be used in ophthalmic applications are needed in order to avoid irreversible BRB disruption.

Ultrasound settings largely influence the efficacy of the method, as well as the safety. The maximum pressure/intensity of the emitted ultrasound wave is one of the critical safety parameters, as it is one of the determining factors for microbubble cavitation regime and the resulting bioeffects. In ophthalmic ultrasound imaging applications, the FDA limits the maximum MI (the ratio of the peak negative pressure over the square root of the transmitted ultrasound frequency) to 0.23 [166]. Looking at the studies that investigated extravasation of molecules from the retinal vasculature, in all cases with successful BRB disruption, an MI higher than 0.23 was used (assuming that the pressures in [81] were given as PNP, as it was not clear from the paper). However, in all three studies, some type of damage was observed in the retina (vasoconstriction of blood vessels, tissue morphological alterations, neuroinflammation, etc.). The only example where an MI within the FDA’s imaging limits was used was from Hirokawa et al. but no extravasation of fluorescein was seen [79]. Considering the intracellular uptake of (drug) molecules, most of the studies report ultrasound intensity (in W/cm^2^) without specifying to which ultrasound intensity definition this corresponds (e.g., spatial peak–temporal peak, spatial peak–pulse average, etc.). For those studies that used the Sonitron 2000 (Rich-Mar, Inola, OK, USA) system, the MI was calculated based on the calibration of the system performed by Kopechek et al. [167]. Sonoda et al. [147] reported successful corneal gene delivery in rabbits in vivo at MI 0.14–0.21 (intensity 0.5–2 W/cm^2^), but when the MI was increased to 0.25 (intensity 3 W/cm^2^), corneal stroma haziness was observed. In addition, Yamashita et al. [103] treated rat eyes with USMB at MI 0.18 (1.2 W/cm^2^) and reported successful gene delivery in conjunctiva without any adverse effects. Overall, it can be concluded that intracellular uptake was successfully enhanced by USMB using MIs within the FDA’s safety limit for imaging applications. In contrast, this approach has not been successful for BRB disruption applications so far, and it is to be investigated whether it can be performed without inducing adverse effects in the retina.

In addition to ultrasound intensity and MI, other ultrasound settings, such as the duty cycle (the ratio of the ultrasound pulse duration to the repetition period) and exposure time (the total time ultrasound waves are transmitted to the target site during treatment), are associated with cell damage and safety issues [32]. Indeed, some of the studies discussed above reported increased cell damage with increasing duty cycle, increasing microbubble concentration and exposure time [83,89,133]. Consequently, a combination of these parameters in addition to the maximum allowed MI is what will likely define the safety limit of USMB in ophthalmology.

Another significant safety aspect is temperature elevation in the eye during ultrasound exposure. Tissue heating depends on the energy of the ultrasound wave and the ability of the tissue to absorb this energy and convert it into heat. Temperature elevation in the lens and cornea can induce the formation of a cataract and alter the expression of a stress protein in corneal epithelial cells, respectively [168]. The regulatory recommendations by the World Federation of Ultrasound in Medicine and Biology (WFUMB) state that tissue temperature increases >1.5 °C above physiological levels should be avoided in the eye [169]. Kowalczuk et al. measured a temperature increase of 3.7 °C in rat lens and 7.3 °C in ciliary muscle during sonication (intensity 2 W/cm^2^, exposure time 120 s) using a digital thermocouple needle [112]. Physiological temperature was recovered a few seconds after sonication stopped and no alterations in the transparency of the lens were observed for up to a month post-treatment. Due to the differences in the size between rat and human eyes, heat distribution might differ between the two species. Consequently, the thermal effects of USMB in the human eye are yet to be determined. In clinical practice, temperature-related side effects in the anterior eye could be limited by adjusting the geometry of the ultrasound probe in such a way that exposure of the lens to ultrasound is avoided. An example of a therapeutic device with such geometry is used in the treatment of high IOP with ultrasound cycloplasty (EyeOP1, Eye Tech Care, Lyon, France). The piezoelectric transducers are positioned in a ring on the surface of the eye that allows the ultrasound beam to be focused on the ciliary body without interfering with the lens [170,171]. In addition to transducer geometry, the ultrasound settings that induce a therapeutic effect, but no thermal side effects, need to be investigated.

Finally, an adverse immune reaction to USMB treatment can affect the safety of USMB in ocular drug delivery. In prior studies, USMB-mediated BBB disruption was associated with inflammatory response, presence of albumin in the brain parenchyma and infiltration of macrophages [172,173]. Although moderate gliosis in the retinal Müller cells can be advantageous in the secretion of neurotrophic factors, prolonged disruption of the BRB might induce severe gliosis, which is associated with uncontrolled cell proliferation and depolarization [174]. Furthermore, the presence of fibrinogen and erythrocytes in the retina as a result of BRB disruption is an undesired potential side effect of USMB treatment. Indeed, some evidence of these adverse effects was observed in earlier BRB-related studies [80,81].

To conclude, in vitro and in vivo studies in animals indicate that USMB can be used safely to improve drug or gene delivery in the eye. However, long-term toxicity studies are required to determine the exact treatment protocol that permits safe translation of this method to the clinic. 

## 6. Future Directions

**Figure 7:**
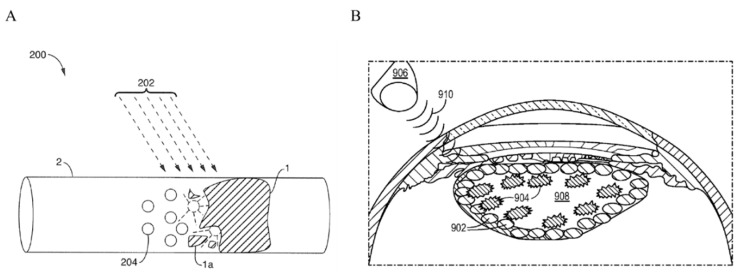
Examples of inventions published as patent applications or patents proposing the use of USMB for the treatment of ocular diseases. (**A**) Sonothrombolysis of a blood clot in a retinal blood vessel. Microbubbles (204) undergo mechanical cavitation when triggered by an externally applied ultrasound beam (202). Oscillations of microbubbles cause the blood clot (1) in the retinal blood vessel (2) to dissolve (1a). Adapted from [180]. (**B**) Fragmentation of cataract using microbubbles. Microbubbles (902) are injected using a thin needle into the lens (908). Ultrasound waves (910) generated by an external source (906) cause the microbubbles to cavitate and fragment the cataracts (904). Adapted from [181].

In addition to the preclinical studies available in the literature, some patents and patent applications have been published, highlighting the potential of USMB in ocular drug delivery in the clinic. A recent patent application by Carpentier et al. describes the technical aspects of an ultrasound device that can be used for targeted drug delivery in the human retina [175]. Another example of microbubble activity against ocular pathologies is the enhancement of blood clot lysis (so-called “sonothrombolysis”). Sonothrombolysis has been investigated in vitro and in vivo as a method to accelerate recanalization after vascular thrombosis in ischemic stroke and heart disease [176,177,178,179]. Utilizing the same principles, Fawzi et al. in 2014 applied for a patent that describes the use of microbubble cavitation against retinal vein occlusions (Figure 7A) [180]. The use of mechanical activity of microbubbles against cataract was described in a recent patent application by Grubbs et al. (Figure 7B) [181]. Microbubbles are injected using a thin needle tip into the lens after capsulorhexis and hydrodissection of the lens. By externally applied ultrasound, microbubble cavitation is triggered, which induces cataract fragmentation. A standard irrigation–aspiration device can then be used for aspiration of the cataract fragments.

## 7. Conclusions

Various studies have investigated the use of USMB as a method to improve drug and gene delivery in ophthalmology. Applications include transient and reversible disruption of the BRB, and enhanced intracellular uptake of anticancer drugs, genes and nanoparticles. The studies discussed in this review report limited side effects caused by USMB in vitro and in vivo in animals. Efficacy and safety are highly dependent on the characteristics of the ultrasound field and the behavior of microbubbles (stable or inertial cavitation) during the treatment. Research into USMB-based permeabilization of the BRB and intracellular delivery of drugs could potentially lead to breakthroughs in the field of ocular therapeutics, allowing for administration of therapeutics in the eye that previously would not be properly delivered and, therefore, suffer from poor efficacy.

## Abbreviations

AMD	age-related macular degeneration
BBB	blood–brain barrier
BLs	bubble liposomes
BRB	blood–retina barrier
CEUS	contrast-enhanced ultrasound
CMB	cationic microbubbles
DPPC	1,2-dipalmitoyl-sn-glycero-3-phosphocholine
DSPA	1,2-distearoyl-snglycero-phosphoacid
DSPC	1,2-distearoyl-sn-glycero-phosphatidylcholine
DSPE-PEG(2k)-OMe	1,2-distearoyl-sn-glycero-3-phosphoethanolamine-N-[methoxy(polyethylene glycol)-2000]
DR	diabetic retinopathy
EGFP	enhanced green fluorescent protein
ES	endostatin
FDA	Food and Drug Administration
F-VEP	flash visual evoked potential
Gd	gadolinium
GFAP	glial fibrillary acidic protein
GFP	green fluorescence protein
H&E	hematoxylin and eosin
iBRB	inner blood–retina barrier
IgG	immunoglobulin G
IgM	immunoglobulin M
ILM	inner limiting membrane
INL	inner nuclear layer
IOP	intraocular pressure
L/siRNA	lipofectamine-formulated siRNA
MFI	mean fluorescence intensity
MI	mechanical index
mNGF	mouse neuron growth factor
mRNA	messenger RNA
mPEG-PLGA-PLL	monomethoxypoly(ethylene glycol)-poly(lactic-co-glycolic acid)-poly L-lysine
M_W_	molecular weight
NMB	neutral microbubbles
NBs	nanobubbles
oBRB	outer blood–retina barrier
OLM	outer limiting membrane
ONL	outer nuclear layer
PDGF	platelet-derived growth factor
pDNA	plasmid DNA
pEGFP	plasmid enhanced green fluorescence protein
PEI	polyethylenimine
PFP	perfluoropropane
PNP	peak-negative pressure
PVR	proliferative vitreoretinopathy
rAAV	recombinant adeno-associated virus
RB	retinoblastoma
RGC	retinal ganglion cells
RP	retinitis pigmentosa
RPE	retina pigment epithelium
siRNA	small interfering RNA
TGF-β2	transforming growth factor-β2
UBM	ultrasound biomicroscopy
USMB	ultrasound and microbubbles
USNB	ultrasound and nanobubbles
USBL	ultrasound and bubble liposomes
VEGF	vascular endothelial growth factor
WFUMB	World Federation of Ultrasound in Medicine and Biology
